# An improved multi-view spectral clustering based on tissue-like P systems

**DOI:** 10.1038/s41598-022-20358-6

**Published:** 2022-11-03

**Authors:** Huijian Chen, Xiyu Liu

**Affiliations:** Shandong Normal University, Business School, Jinan, 250385 China

**Keywords:** Mathematics and computing, Computer science, Information technology, Computational biology and bioinformatics, Data mining, Machine learning

## Abstract

Multi-view spectral clustering is one of the multi-view clustering methods widely studied by numerous scholars. The first step of multi-view spectral clustering is to construct the similarity matrix of each view. Consequently, the clustering performance will be greatly affected by the quality of the similarity matrix of each view. To solve this problem well, an improved multi-view spectral clustering based on tissue-like P systems is proposed in this paper. The optimal per-view similarity matrix is generated in an iterative manner. In addition, spectral clustering is combined with the symmetric nonnegative matrix factorization method to directly output the clustering results to avoid the secondary operation, such as k-means or spectral rotation. Furthermore, improved multi-view spectral clustering is integrated with the tissue-like P system to enhance the computational efficiency of the multi-view clustering algorithm. Extensive experiments verify the effectiveness of this algorithm over other state-of-the-art algorithms.

## Introduction

In 1998, membrane computing^1^ (also known as the P system or membrane system) was first proposed by Pânu, an academician of the European Academy of Sciences and the Romanian Academy of Sciences. As a branch of natural computing, membrane computing is a distributed and parallel computing model abstracted from the structure and function of biological cells and the collaboration of cell groups such as organs and tissues. P system consists of three parts: membrane structure, multiple sets of objects and rules. Up to now, membrane computing mainly includes three basic computational models: cell-like P system^2^, tissue-like P system^3,4^ and nerve-like P system^5,6^. Tissue-like P system was proposed inspired by the collaboration between organs and cells in tissues, which can be described by a arbitrary graph. The nodes in the graph correspond to cells (the environment is regarded as a specific node), and the edges correspond to the communication channels between cells. If there is a side between the two nodes, it means that the corresponding cells can communicate through rules. Researchers’ research on membrane computing is mainly divided into theoretical research and application. In terms of theoretical research, numerous variants of membrane systems have been proposed by researchers. Luo et al.^7^ proposed a tissue-like P systems with evolutionary codirection / reverse rules. Objects would be changed in the transmission process, and the assumption that the number of objects in the environment is infinite was removed, which reduced the impact of the environment on the system and made the environment no longer provide powerful energy for cells. Luo et al.^8^ proposed an homeostasis tissue-like P system, which assumed that the environment no longer provided energy for cells, and introduced multiple set rewriting rules in tissue-like P system. In terms of application of tissue-like P system, the uncertainty and computational parallelism of P system make it possible to combine with other algorithms to improve computational efficiency. Jiang et al.^9^ introduced a tissue-like P system with active membrane to improve the clustering algorithm, which could improve the efficiency of the algorithm and reduced the computational complexity.

With the rapid development of multimedia technology, multi-view data appears in large numbers, which means that the same object can be described from different angles. For example, a person can be photographed from different angles, each of which corresponds to a view. A piece of news can be broadcast on television or presented in words. These are kind of different views. Such data is considered multi-view data^10,11^. The application of multi-view learning in clustering problems produces a great deal multi-view clustering algorithm suitable for multi-view data. Multi-view clustering aims to classify similar data points into the same cluster and search for consistent clustering results in different views by combining multiple available feature information, so as to divide different types of points into different clusters. Multi-view clustering^12–14^ can be roughly divided into several types, such as multi-view subspace clustering^15,16^, multi-view spectral clustering^17–19,29^, multi-view K-means clustering^20^, etc. Multi-view spectral clustering has been widely studied for its ability to better process nonlinear data. Multi-view spectral clustering requires three separate steps: (1) the similarity matrix of each view is constructed. (2) all the similarity matrices are fused and the spectral embedding matrix is obtained. (3) k-means or spectral rotation operation is performed on spectral embedding matrix to get clustering results. The construction of the similarity matrix of each view is the first step, consequently, the quality of the similarity matrix will affect the clustering performance. Nevertheless, the existing multi-view spectral clustering algorithms do not obtain the affinity matrix of each view in the light of the characteristics and quality of each view, but in a static way. On the other hand, the post-processing operation of multi-view spectral clustering will lose momentous information, which will also exert influence on the clustering performance.

**Figure 1:**
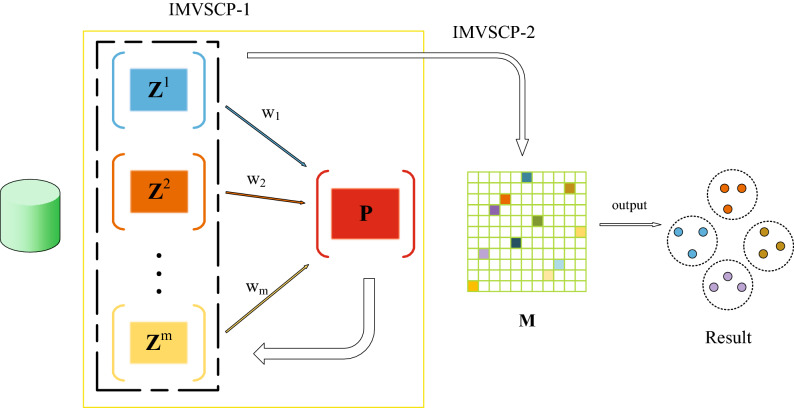
The basic process of IMVSCP algorithm is as follows: The IMVSCP algorithm is divided into two sub-algorithms, one is the IMVSCP-1 algorithm, and the other is the IMVSCP-2 algorithm. The IMVSCP-1 algorithm firstly obtains the similarity matrix $\left( {{{Z}^v}} \right) _{v = 1}^m$ by K-nearest neighbor algorithm, and then fuses $\left( {{{Z}^v}} \right) _{v = 1}^m$ into a unified matrix *P* by weighted fusion operation. In turn, the unified matrix *P* updates the similarity matrix for each view. IMVSCP-1 algorithm is an iterative updating process. The IMVSCP-2 algorithm takes the updated similarity matrix $\left( {{{Z}^v}} \right) _{v = 1}^m$ obtained by the IMVSCP-1 algorithm as input, and combines the spectral clustering algorithm and symmetric non-negative matrix factorization algorithm to obtain the non-negative embedding matrix *M*, so as to directly output the clustering results.

To solve these problems, an improved multi-view spectral clustering algorithm based on tissue-like P systems (IMVSCP) is proposed in this paper. The optimized similarity matrix for each view is constructed in a weighted iterative manner. In addition, the discrete nonnegative embedding matrix is obtained by combining with the symmetric nonnegative matrix factorization method to directly output the clustering results, avoiding the influence of post-processing. Furthermore, the improved multi-view graph clustering algorithm is combined with the tissue-like P system to improve the efficiency of the algorithm. Figure 1 displays the flow of the improved multi-view spectral clustering algorithm. The main contributions of this paper are summarized as follows:

- In order to fully utilize the features of each view, the method of acquiring the similarity matrix of each view is optimized. Instead of getting the affinity matrix of each view statically, a dynamic weighted iterative method is adopted to obtain the optimal similarity matrix of each view to improve the clustering performance.
- The post-processing of multi-view spectral clustering will lead to the loss of significant information. Therefore, multi-view spectral clustering is combined with symmetric nonnegative matrix factorization method to obtain discrete nonnegative embedding matrix and output the clustering results directly.
- Due to the computational parallelism of tissue-like P system, the improved multi-view spectral clustering algorithm is embedded in the framework of tissue-like P system to improve the computational efficiency of the algorithm.
- Extensive experiments have been conducted to verify that IMVSCP algorithm can achieve better clustering performance compared to the state-of-the-art algorithms.

The structure of this paper is as follows. In section “Related work”, we summarize the related work of multi-view spectral clustering and tissue-like P system, and we describe the improved multi-view spectral clustering algorithm and the initial configuration of tissue-like P system in detail in section “The proposed method”. In section “Experiments”, experiments are carried out to verify the effectiveness of the algorithm. We discuss the experimental results and the shortcomings of the proposed algorithm in section “Discussion”. In section “Conclusion”, we summarize this work.

## Related work

### Multi-view clustering

Multi-view graph clustering and multi-view spectral clustering complete the clustering process by means of exploring the local geometric structure of data. A great deal of scholars has carried out relevant studies with the purpose of learning the similarity graph of each view from the original data better. Li et al.^21^ constructed the similarity graph in the embedded space instead of the original space to deal with noise excellently and learn high-quality similarity graph. Zhang et al.^22^ proposed flexible multi-view unsupervised graph embedding (FMUGE), which introduced a flexible regression residual term to relax the strict linear mapping. New-coming data and noise were better processed, and the original data negotiated with the learned low-dimensional representation in the process. In order to ensure the consistency between multiple views, FMUGE adaptively weighted and fused different features to obtain the optimal similarity graph of multi-view consistency. In order to better mine view-specific information, Shi et al.^23^ proposed self-weighting multi-view spectral clustering based on nuclear norm (SMSC-NN) and introduced the nuclear norm to perform sparse processing on the obtained unified similarity matrix to make it better oriented to spectral clustering. The post-processing procedure required to obtain the clustering results will lose useful information. Wang et al.^24^ proposed two parameter-free weighted multi-view projected clustering methods, which simultaneously performed structured graph learning and dimensionality reduction and could directly utilize the obtained structured graph to extract clustering indicators without other discretization processes like previous graph-based clustering methods. Nie et al.^25^ proposed self-weighted multiview clustering with multiple Graphs (SwMC). Once the target graph is acquired in the model, SwMC can directly assign cluster labels to each data point without any post-processing.

### Tissue-like P system

The tissue-like P system consists of cells and environment, and carries out the evolution and transmission of objects through rules. In this paper, we introduce the formal definition of tissue-like P system with rule triggering mechanism:

$$\begin{aligned} \Pi \mathrm{{ = (}}O,{\sigma _1},{\sigma _2}, \cdots ,{\sigma _m},syn,{i_0},E\mathrm{{)}}. \end{aligned}$$

where

1. $\textit{O}$ is a finite multiset of objects.
2. $syn \subseteq \left\{ {1,2, \cdots ,m} \right\} \times \left\{ {1,2, \cdots ,m} \right\}$ represents communication channels between cells.
3. ${i_{0}}$ is the output cell.
4. *E* represents any number of copies of objects in the environment.
5. ${\sigma _{1}},{\sigma _{2}}, \cdots ,{\sigma _{m}}$ represents m cells, and ${\sigma _{i}}$ is defined as follows:
  $$\begin{aligned} {\sigma _{i}} = \left( {{w_{i}},{R_{i}}} \right) \end{aligned}$$
  where ${w_{i}}$ is the initial state of cell *i*. ${R_{i}}$ represents a finite set of rules in cell *i* including rule with triggering mechanisms: when the condition $\varepsilon$ is satisfied, the rule is triggered and executed preferentially.

## The proposed method

### Initializing the similarity matrix for each view

For raw data $\left( {{{\varvec{X}}^v}} \right) _{v = 1}^m \in {\mathbb {{R}}^{{d_i} \times n}}$, where ${d_i}$ is the dimension of the ith view, *m* and *n* are the number of views and the number of data points respectively, each view is initialized to get its affinity matrix $\left( {{{\varvec{Z}}^v}} \right) _{v = 1}^m \in {\mathbb {{R}}^{n \times n}}$. Greater similarity should be given to two similar data points, while smaller similarity should be given when two data points are far apart^26^. Therefore, we specify that the objective function of initializing the similarity matrix is:

1 $$\begin{aligned} \begin{array}{l} \mathop {\min }\limits _{\{ {{Z}^v}\} } \sum \limits _{v = 1}^m {\sum \limits _{i,j = 1}^n {\parallel {x}_i^v - {x}_j^v\parallel _2^2z_{ij}^v + \alpha \sum \limits _{v = 1}^m {\sum \limits _{i = 1}^n {\parallel {z}_i^v\parallel _2^2} } } } \\ s.t.\;\forall v,z_{ii}^v = 0,z_{ij}^v \ge 0,{{1}^T}{z}_i^v = 1. \end{array} \end{aligned}$$

According to reference^26^, Eq. (2) is obtained by optimizing Eq. (1) to initialize the similarity matrix of each view.

2 $$\begin{aligned} {\hat{z}}_{ij}^v = \left\{ {\begin{array}{*{20}{c}} {\frac{{{q_{i,e + 1}} - {q_{ij}}}}{{e{q_{i,e + 1}} - \sum \nolimits _{h = 1}^e {{q_{ih}}} }}}&{}{j \le e}\\ 0&{}{j > e}, \end{array}} \right. \end{aligned}$$

where ${q_{i,j}} = \parallel {\varvec{x}}_i^v - {\varvec{x}}_j^v\parallel _2^2$, *e* is the number of neighbors.

### Optimizing the similarity matrix for each view

The initial similarity matrix $\left( {{{Z}^v}} \right) _{v = 1}^{m}$ are fused to obtain a unified matrix *P* so as to update the similarity matrix of each view iteratively. We compute the unified matrix *P* by the following formula:

3 $$\begin{aligned} \begin{array}{l} \mathop {\min }\limits _{P} \sum \limits _{v = 1}^m {{w_v}\parallel {P} - {{Z}^v}\parallel _F^2} \\ s.t.\,\forall i,{p_{ij}} \ge 0,{{1}^T}{{p}_i} = 1, \end{array} \end{aligned}$$

where ${w_v}$ is the weight of the *v* views, the formula is:

4 $$\begin{aligned} {w_v} = \frac{1}{{2\sqrt{\parallel {P} - {{Z}^v}\parallel _F^2} }}. \end{aligned}$$

Due to the different quality of each view, the contribution to the clustering result is not the same. Therefore, each view needs to be assigned a different weight, with high quality views being given a larger weight and low quality views being given a smaller weight. As can be seen from Eq. (4), ${w_v}$ is related to unified matrix *P* and the similarity matrix $\left( {{{Z}^v}} \right) _{v = 1}^m$, so ${w_v}$ can be automatically iterated in the update without any trivial solution. Therefore, Eq. (3) avoids the appearance of hyperparameters. Next, we combine Eqs. 1 and 3 to update $\left( {{{Z}^v}} \right) _{v = 1}^m$ with the unified matrix *P*:

5 $$\begin{aligned} \begin{array}{l} \mathop {\min }\limits _{\{ {{Z}^v}\} } \sum \limits _{v = 1}^m {\sum \limits _{i,j = 1}^n {\parallel {x}_i^v - {x}_j^v\parallel _2^2z_{ij}^v + \alpha \sum \limits _{v = 1}^m {\sum \limits _{i = 1}^n {\parallel {z}_i^v\parallel _2^2} } } } \\ \quad \,\, + \sum \limits _{v = 1}^m {{w_v}\parallel {P} - {{Z}^v}\parallel _F^2} \\ s.t.\,\forall v,z_{ii}^v = 0,z_{ij}^v \ge 0,{{1}^T}{z}_i^v = 1,\\ \quad \quad \,{p_{ij}} \ge 0,{{1}^T}{{p}_i} = 1. \end{array} \end{aligned}$$

We impose rank constraint on the Laplacian matrix of the unified matrix *P* to make the optimized $\left( {{{Z}^v}} \right) _{v = 1}^m$ more suitable for clustering problem. The Laplacian matrix of matrix *P* is defined as ${{L}_P} = {{D}_P} - {{\left( {{{P}^T} + {P}} \right) } /2}$, where the degree matrix is a diagonal matrix whose i-th diagonal element is $\sum \nolimits _j {{{\left( {{p_{ij}} + {p_{ji}}} \right) } /2}}$. Here we introduce Theorem 1:

#### Theorem 1

The multiplicity *c* of the eigenvalue 0 of the Laplacian matrix ${{L}_P}$ is equal to the number of connected components of the graph of ${{L}_P}$.

It can be seen from Theorem 1 that the unified matrix *P* obtained when we set $\mathrm{{rank}}\left( {{{L}_P}} \right) = n - c$ can be divided into *c* clusters, which ensures that the optimized $\left( {{{Z}^v}} \right) _{v = 1}^m$ can better handle the clustering problem. Therefore, Eq. (5) can be transformed into the following formula:

6 $$\begin{aligned} \begin{array}{l} \mathop {\min }\limits _{\{ {{Z}^v}\} } \sum \limits _{v = 1}^m {\sum \limits _{i,j = 1}^n {\parallel {x}_i^v - {x}_j^v\parallel _2^2z_{ij}^v + \alpha \sum \limits _{v = 1}^m {\sum \limits _{i = 1}^n {\parallel {z}_i^v\parallel _2^2} } } } \\ \quad \;\; + \sum \limits _{v = 1}^m {{w_v}\parallel {P} - {{Z}^v}\parallel _F^2} \\ s.t.\;\forall v,z_{ii}^v = 0,z_{ij}^v \ge 0,{{1}^T}{z}_i^v = 1,\\ \quad \quad \;{p_{ij}} \ge 0,{{1}^T}{{p}_i} = 1,rank\left( {{{L}_P}} \right) = n - c. \end{array} \end{aligned}$$

It is very difficult to solve problem 6 directly, so according to Ky Fan’s Theorem^25^ we have:

7 $$\begin{aligned} \sum \limits _{i = 1}^c {{\ell _i}\left( {{{L}_P}} \right) = \mathop {\min }\limits _{{{F}_1} \in {^{n \times c}}} Tr\left( {{F}_1^T{{L}_P}{{F}_1}} \right) }, \end{aligned}$$

where ${{F}_1}$ is the spectral embedding matrix and ${\ell _i}\left( {{{L}_P}} \right)$ represents the i-th smallest eigenvalue of ${{L}_P}$. Then, Eq. (6) becomes:

8 $$\begin{aligned} \begin{array}{l} \mathop {\min }\limits _{\{ {{Z}^v}\} } \sum \limits _{v = 1}^m {\sum \limits _{i,j = 1}^n {\parallel {x}_i^v - {x}_j^v\parallel _2^2z_{ij}^v + \alpha \sum \limits _{v = 1}^m {\sum \limits _{i = 1}^n {\parallel {z}_i^v\parallel _2^2} } } } \\ \quad \;\; + \sum \limits _{v = 1}^m {{w_v}\parallel {P} - {{Z}^v}\parallel _F^2} + 2\phi Tr\left( {{F}_1^T{{L}_P}{{F}_1}} \right) \\ s.t.\;\forall v,z_{ii}^v = 0,z_{ij}^v \ge 0,{{1}^T}{z}_i^v = 1,\\ \quad \quad \;{p_{ij}} \ge 0,{{1}^T}{{p}_i} = 1,{F}_1^T{{F}_1} = {I}, \end{array} \end{aligned}$$

where $\phi$ is a parameter that can be adjusted automatically. Next, we get the optimal $\left( {{{Z}^v}} \right) _{v = 1}^m$ by iteratively optimizing Eq. (8). There are four variables in Eq. (8) that need to be optimized.

*Update* $\left( {{{Z}^v}} \right) _{v = 1}^m$ , *fix* ${w_v}$, *P* *and* ${{F}_1}$. When we fix ${w_v}$, *P* and ${{F}_1}$, Eq. (8) transforms into the following form:

9 $$\begin{aligned} \begin{array}{l} \mathop {\min }\limits _{\{ {{Z}^v}\} } \sum \limits _{v = 1}^m {\sum \limits _{i,j = 1}^n {\parallel {x}_i^v - {x}_j^v\parallel _2^2z_{ij}^v + \alpha \sum \limits _{v = 1}^m {\sum \limits _{i = 1}^n {\parallel {z}_i^v\parallel _2^2} } } } \\ \quad \;\; + \sum \limits _{v = 1}^m {{w_v}\parallel {P} - {{Z}^v}\parallel _F^2} \\ s.t.\;\forall v,z_{ii}^v = 0,z_{ij}^v \ge 0,{{1}^T}{z}_i^v = 1. \end{array} \end{aligned}$$

It can be seen from Eq. (9) that updating ${{Z}^v}$ is independent for each view, so we have:

10 $$\begin{aligned} \begin{array}{l} \mathop {\min }\limits _{{{Z}^v}} \sum \limits _{i,j = 1}^n {\parallel {x}_i^v - {x}_j^v\parallel _2^2z_{ij}^v + } \alpha \sum \limits _i^n {\parallel {z}_i^v\parallel _2^2} + {w_v}\parallel {P} - {{Z}^v}\parallel _F^2\\ s.t.\;z_{ii}^v = 0,z_{ij}^v \ge 0,{{1}^T}{z}_i^v = 1. \end{array} \end{aligned}$$

According to reference^28^, we get the solution:

11 $$\begin{aligned} z_{ij}^v = \left\{ {\begin{array}{*{20}{c}} {\frac{{{q_{i,e + 1}} - {q_{ij}} + 2{w_v}{p_{ij}} - 2{w_v}{p_{i,e + 1}}}}{{e{q_{i,e + 1}} - \sum \nolimits _{h = 1}^e {{q_{ih}} - 2e{w_v}{p_{i,e + 1}}} + 2\sum \nolimits _{h = 1}^e {{w_v}{p_{ih}}} }}}&{}{j \le e}\\ 0&{}{j > e}, \end{array}} \right. \end{aligned}$$

where the relevant symbols are the same as those defined in section “Initializing the similarity matrix for each view”. *Update* ${w_v}$, *fix* $\left( {{{Z}^v}} \right) _{v = 1}^m$, *P* *and* ${{F}_1}$. As we know from Eq. (4), the weight of view *v* is determined by the unified matrix *P* and the similarity matrix ${{Z}^v}$. In consequence, when ${{Z}^v}$ and *P* are fixed, ${w_v}$ is updated by Eq. (4). *Update* *P*, *fix* $\left( {{{Z}^v}} \right) _{v = 1}^m$, ${w_v}$ *and* ${{F}_1}$. When $\left( {{{Z}^v}} \right) _{v = 1}^m$, ${w_v}$ and ${{F}_1}$ are fixed and $Tr\left( {{F}_1^T{{L}_P}{{F}_1}} \right) = 1 /2\sum \nolimits _{i,j} {\parallel {{f}_{1i}} - {{f}_{1j}}\parallel _2^2{p_{ij}}}$, Eq. (8) becomes:

12 $$\begin{aligned} \begin{array}{l} \mathop {\min }\limits _{P} \sum \limits _{v = 1}^m {\sum \limits _{i,j = 1}^n {{w_v}{{\left( {{p_{ij}} - z_{ij}^v} \right) }^2}} + \lambda \sum \limits _{i,j = 1}^n {\parallel {{f}_{1i}} - {{f}_{1j}}\parallel _2^2{p_{ij}}} } \\ s.t.\;\forall i,{p_{ij}} \ge 0,{{1}^T}{{p}_i} = 1. \end{array} \end{aligned}$$

It is obvious that the Eq. (12) is independent for each view, in the meantime, we define ${b_{ij}} = \parallel {{f}_{1i}} - {{f}_{1j}}\parallel _2^2$, afterwards, the Eq. (12) is transformed into:

13 $$\begin{aligned} \begin{array}{l} \mathop {\min }\limits _{{p^i}} \sum \limits _{v = 1}^m {\sum \limits _{j = 1}^n {{w_v}{{\left( {{p_{ij}} - z_{ij}^v} \right) }^2}} + \lambda \sum \limits _{j = 1}^n {{b_{ij}}{p_{ij}}} } \\ s.t.\;\forall i,{p_{ij}} \ge 0,{{1}^T}{{p}_i} = 1. \end{array} \end{aligned}$$

According to reference^28^, solving the problem 13 equals solving the problem 14:

14 $$\begin{aligned} \begin{array}{l} \mathop {\min }\limits _{{{p}_i}} \sum \limits _{v = 1}^m {\parallel {{p}_i} - {z}_i^v + \frac{\lambda }{{2m{w_v}}}{{b}_i}\parallel _2^2} \\ s.t.\;\forall i,{p_{ij}} \ge 0,{{1}^T}{{p}_i} = 1, \end{array} \end{aligned}$$

where the j-th element of ${{b}_i}$ is ${b_{ij}}$.The optimal solution of problem 14 is given in reference^28^. ${{F}_1}$, *fix* $\left( {{{Z}^v}} \right) _{v = 1}^m$, ${w_v}$ *and* *P*. When $\left( {{{Z}^v}} \right) _{v = 1}^m$, ${w_v}$ and *P* are fixed, Eq. (8) becomes the following form:

15 $$\begin{aligned} \mathop {\min }\limits _{{{F}_1}} Tr\left( {{F}_1^T{{L}_P}{{F}_1}} \right) ,\;s.t.\;{F}_1^T{{F}_1} = {I}. \end{aligned}$$

The optimal solution of ${{F}_1}$ is composed of the eigenvectors corresponding to the first *c* eigenvalues of ${{L}_P}$.The process of optimizing the similarity matrix of each view is explained by algorithm 1 as a whole.
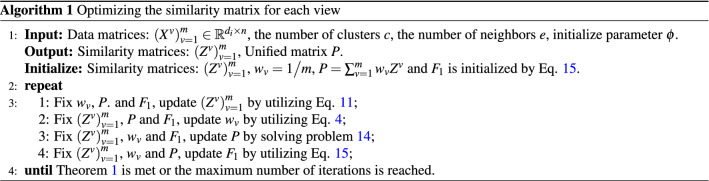


### Improved multi-view spectral clustering

In this section, spectral clustering is combined with symmetric nonnegative matrix factorization method (symNMF)^29^ to directly output clustering results. The relationship between spectral clustering and symNMF requires to be comprehended. The objective function of spectral clustering is:

16 $$\begin{aligned} \mathop {\min }\limits _{{{F}^T}{F} = {I}} Tr\left( {{{F}^T}{{D}^{ - {1} /2}}}{{L}_Z}{{D}^{ - {1 /2}}}{F} \right) , \end{aligned}$$

where *Z* is the similarity matrix, *D* is the degree matrix, ${{L}_Z}$ is the Laplacian matrix of *Z*, and *F* is the spectral embedding matrix. Since ${{L}_Z} = {D} - {S}$, Eq. (16) becomes:

17 $$\begin{aligned} \mathop {\max }\limits _{{{F}^T}{F} = {I}} Tr\left( {{{F}^T}{{D}^{ - {1 / 2}}}{Z}{{D}^{ - {1 /2}}}{F}} \right) . \end{aligned}$$

The optimized spectral embedding matrix *F* is acquired by utilizing Eq. (17), and then k-means or spectral rotation operation is performed on it to obtain clustering results. The symNMF method is introduced below. For a matrix *Z*, its symNMF objective function is:

18 $$\begin{aligned} \begin{array}{l} \mathop {\min }\limits _{{M} \in {{\mathbb {{R}}}^{n \times c}}} \parallel {Z} - {M}{{M}^T}\parallel _F^2\\ s.t.\;{M} \ge 0. \end{array} \end{aligned}$$

Equation 18 can be converted to the following form:

19 $$\begin{aligned} \begin{array}{l} \mathop {\min }\limits _{M} Tr\left( {{M}{{M}^T}{M}{{M}^T}} \right) - 2Tr\left( {{{M}^T}{ZM}} \right) \\ \quad \quad s.t.\;{M} \ge 0. \end{array} \end{aligned}$$

When we change the constraint ${M} \ge 0$ to ${{M}^T}{M} = {I}$ and the matrix *Z* to ${{D}^{ - {1 / 2}}}{Z}{{D}^{ - {1/2}}}$, the Eq. (19) becomes the following form:

20 $$\begin{aligned} \begin{array}{l} \mathop {\max }\limits _{M} Tr\left( {{{M}^T}{{D}^{ - {1 / 2}}}{Z}{{D}^{ - {1 / 2}}}{M}} \right) \\ \quad \quad s.t.\;{{M}^T}{M} = {I}. \end{array} \end{aligned}$$

It is obvious that Eq. (20) is consistent with the objective function Eq. (17) of spectral clustering. We extend this connection to the multi-view spectral clustering, so as to give the improved multi-view spectral clustering objective function proposed in this paper:

21 $$\begin{aligned} \begin{array}{l} \mathop {\min }\limits _{\left\{ {{F}_2^v} \right\} _{v = 1}^m,{M}} \sum \limits _{v = 1}^m {{\alpha ^v}\parallel {{Z}^v} - {M}{{\left( {{F}_2^v} \right) }^T}\parallel _F^2} \\ \quad \mathrm{{s}}\mathrm{{.t}}\mathrm{{.}}\;{M} \ge 0,{\left( {{F}_2^v} \right) ^T}{F}_2^v = {I}, \end{array} \end{aligned}$$

where

22 $$\begin{aligned} {\alpha ^v} = \frac{1}{{\parallel {{Z}^v} - {M}{{\left( {{F}_2^v} \right) }^T}{\parallel _F}}}, \end{aligned}$$

where ${F}_2^v$ is the spectral embedding matrix of the v-th view, and *M* is a consistent nonnegative embedding matrix, and the cluster corresponding to the maximum value of each row is the cluster to which the data point belongs. Therefore, clustering results can be directly given. It is worth noting that ${{Z}^v}$ is optimized by iterative update of algorithm 1. In addition, the weight of the v-th view can be automatically determined by ${\alpha ^v}$ based on ${{Z}^v}$ and ${F}_2^v$.

Equation 21 has two variables to be optimized. Next, we optimize Eq. (21) by iterative method: Fix ${F}_2^v$, *Update* *M*. By means of fixing ${F}_2^v$ and removing irrelevant variables, the following problem is solved to optimize *M*:

23 $$\begin{aligned} \begin{array}{l} \mathop {\min }\limits _{M} \sum \limits _{v = 1}^m {{\alpha ^v}\parallel {{Z}^v} - {M}{{\left( {{F}_2^v} \right) }^T}\parallel _F^2} \\ \quad \mathrm{{s}}\mathrm{{.t}}\mathrm{{.}}\;{M} \ge 0. \end{array} \end{aligned}$$

Since ${\left( {{F}_2^v} \right) ^T}{F}_2^v = {I}$, Eq. (23) becomes:

24 $$\begin{aligned} \begin{array}{l} \mathop {\min }\limits _{M} \sum \limits _{v = 1}^m {{\alpha ^v}\parallel {{Z}^v}{F}_2^v - {M}\parallel _F^2} \\ \quad \mathrm{{s}}\mathrm{{.t}}\mathrm{{.}}\;{M} \ge 0. \end{array} \end{aligned}$$

Furthermore, by introducing and removing fixed terms, the Eq. (24) is changed into:

25 $$\begin{aligned} \begin{array}{l} \mathop {\min }\limits _{M} \parallel \sum \limits _{v = 1}^m {{\alpha ^v}{{Z}^v}{F}_2^v - {M}\parallel _F^2} \\ \quad \mathrm{{s}}\mathrm{{.t}}\mathrm{{.}}\;{M} \ge 0, \end{array} \end{aligned}$$

where

26 $$\begin{aligned} {{\hat{\alpha }} ^v} = \frac{{{\alpha ^v}}}{{\sum \nolimits _{v = 1}^m {{\alpha ^v}} }}. \end{aligned}$$

The optimal solution of Eq. (25) is:

27 $$\begin{aligned} {{M}^*} = \max ({0},\sum \nolimits _{v = 1}^m {{\alpha ^v}{{Z}^v}{F}_2^v} ). \end{aligned}$$

*Fix* *M*, *Update* ${F}_2^v$. When *M* is fixed, ${F}_2^v$ is independent for each view. In consequence, ${F}_2^v$ is updated by solving the following problem:

28 $$\begin{aligned} \begin{array}{l} \mathop {\min }\limits _{{F}_2^v} \parallel {{Z}^v} - {M}{\left( {{F}_2^v} \right) ^T}\parallel _F^2\\ \;s.t.\;{\left( {{F}_2^v} \right) ^T}{F}_2^v = {I}. \end{array} \end{aligned}$$

Singular value decomposition of ${\left( {{{Z}^v}} \right) ^T}{M}$ yields left singular vector *U* and right singular vector *V*. The optimal solution of Eq. (28) is:

29 $$\begin{aligned} {F}_2^v = {U}{{V}^T}. \end{aligned}$$

Algorithm 2 illustrates the process of improved multi-view spectral clustering.
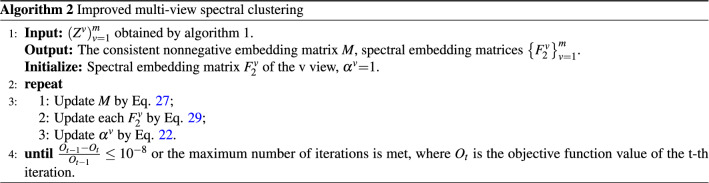


### Initial configuration of tissue-like P system

**Figure 2:**
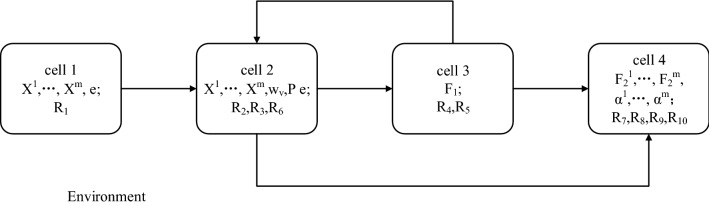
The basic framework of tissue-like P system.

Before the calculation, the initial configuration of tissue-like P system utilized in this paper is described. Figure 2 shows the basic framework of tissue-like P system. This type of tissue-like P system has four cells, with arrows representing channels between cells where objects can be transmitted in one direction. Outside the cell is the environment. First of all, several rules are defined as follows:

- $R_1$: Eq. (2) is utilized to get initialized $\left( {{{Z}^v}} \right) _{v = 1}^m$.
- $R_2$: Eq. (11) is utilized to update $\left( {{{Z}^v}} \right) _{v = 1}^m$ and send it to cell 3.
- $R_3$: Eq. (4) is utilized to update ${w_v}$ and send it to cell 3.
- $R_4$: Eq. (14) is utilized to update *P* and send its copy to cell 2.
- $R_5$: Eq. (15) is utilized to update ${{F}_1}$.
- $R_6$(Trigger mechanism rule): $\left( {{{Z}^v}} \right) _{v = 1}^m$ are sent to cell 4 when theorem 1 is met or the maximum number of iterations is reached.
- $R_7$: Eq. (27) is utilized to update *M*.
- $R_8$: Eq. (29) is utilized to update $\left\{ {{F}_2^v} \right\} _{v = 1}^m$.
- $R_9$: Eq. (22) is utilized to update $\left\{ {{\alpha ^v}} \right\} _{v = 1}^m$.
- $R_{10}$(Trigger mechanism rule): *M* is output when $\frac{{{O_{t - 1}} - {O_t}}}{{{O_{t - 1}}}} \le {10^{ - 8}}$ or the maximum number of iterations is met.

It is worth noting that there is a priority relationship between these rules. Rules with higher priorities are executed before rules with lower priorities. The priorities of rules are as follows:

$$R_2 \rightarrow R_3$$; $$R_4 \rightarrow R_5$$; $$R_7 \rightarrow R_8 \rightarrow R_9 \rightarrow R_7$$

The priority of the rule decreases with the direction of the arrow, and it should be noted that the three rules $R_7 - R_9$ are executed according to priority and loop. Next, the initial configuration of the tissue-like P system is presented:

- cell 1: $$\left( {{{X}^v}} \right) _{v = 1}^m$$, *e*; $$R_1$$.
- cell 2: $$\left( {{{X}^v}} \right) _{v = 1}^m$$, $${w_v}$$, *P*, *e*; $$R_2, R_3, R_6$$.
- cell 3: $${{F}_1}$$; $$R_4, R_5$$.
- cell 4: $$\left\{ {{F}_2^v} \right\} _{v = 1}^m$$, $$\left\{ {{\alpha ^v}} \right\} _{v = 1}^m$$; $$R_7, R_8, R_9, R_{10}$$.

### The calculation procedure

In this section, the calculation process of the improved multi-view spectral clustering algorithm in the tissue-like P system is illustrated in detail.

- Step 1: Rule $R_1$ in cell 1 is executed to initialize the similarity matrices $\left( {{{Z}^v}} \right) _{v = 1}^m$.
- Step 2: Rule $R_2$ in cell 2 is executed to optimize the similarity matrices $\left( {{{Z}^v}} \right) _{v = 1}^m$ and transmit them to cell 3.
- Step 3: Rule $R_3$ in cell 2 is executed to optimize the weight ${w_v}$ and transmit them to cell 3.
- Step 4: Rule $R_4$ in cell 3 is executed to optimize the unified matrix *P* and transmit its copy to cell 2.
- Step 5: Rule $R_5$ in cell 3 is executed to optimize the spectral embedding matrix ${{F}_1}$.

Steps 2-5 constitute an iterative process.

- Step 6: When the trigger condition for rule $R_6$ in cell 2 is triggered, $R_6$ is executed to transfer $\left( {{{Z}^v}} \right) _{v = 1}^m$ to cell 4.
- Step 7: Rule $R_7$ is executed to optimize the nonnegative embedding matrix *M* after cell 4 receives $\left( {{{Z}^v}} \right) _{v = 1}^m$ from cell 3.
- Step 8: Rule $R_8$ in cell 4 is executed to optimize the spectral embedding matrices $\left\{ {{F}_2^v} \right\} _{v = 1}^m$.
- Step 9: Rule $R_9$ in cell 4 is executed to optimize $\left\{ {{\alpha ^v}} \right\} _{v = 1}^m$.

Steps 7-9 loop.

- When the trigger condition of $R_{10}$ in cell 4 is triggered, $R_{10}$ is executed to output *M*. The calculation terminates.

## Experiments

### Evaluate indicators and datasets

In this section, relevant experiments were carried out to verify the effectiveness of IMVSCP algorithm. Six evaluation indicators were selected: Acc (Accuracy), NMI (Normalized Mutual Information), Recall, Precision, F-score, and ARI (Adjusted Rand Index). These indicators are widely utilized to evaluate the clustering performance of multi-view clustering, and their definitions are seen in reference^31^. The larger the index value is, the better the clustering performance is. Six datasets were used as supporting datasets in this paper, the specific information is as follows:

**BBCSport **^32^: This is a text dataset with 500 samples and has two views with dimensions 3183 and 3203. It can be divided into five categories.

**ORL **^33^: The dataset is an image dataset, which contains 400 face images from 40 individuals. There are four views, whose dimensions are: 512, 59, 864, 254.

**MSRC **^34^: The dataset is an image dataset that contains 210 objects from seven categories and has five features to describe: CM, GIST, CENT, HOG, and LBP, and their dimensions are as follows:24, 576, 512, 256, 254.

**Mfeat:** This is a handwritten dataset with 2000 objects, which contains 10 digits. In this paper, three views were utilized for this dataset, whose dimensions are: 76, 216, 64.

**NUS **^35^: This is a real image dataset of 2400 samples, described by six features of dimension 64, 144, 73, 128, 225 and 500, divided into 12 categories.

**3-sources **^36^: 3-sources is a text dataset of news stories from three news companies (views): BBC, Reuters and The Guardian. The dimensions of the three views are 3560, 3631, 3068 respectively. 169 samples are divided into six categories.

Table 1 shows the information for the dataset, where *di* is the dimension of the i-th view.

**Table 1:** Information about six datasets.

Datasets	BBCSport	ORL	MSRC	Mfeat	NUS	3-sources
*d*1	3183	512	24	76	64	3560
*d*2	3203	59	576	216	144	3631
*d*3	–	864	512	64	73	3068
*d*4	–	254	256	−	128	−
*d*5	–	–	254	–	225	–
*d*6	–	–	–	–	500	–
*Sample*	544	400	210	2000	2400	169
*cluster*	5	40	7	10	12	6

### Clustering results

In order to compare the effectiveness of IMVSCP algorithm, ten comparison algorithms were used in this experiment, among which the first one was single-view clustering algorithm and the remaining nine were multi-view clustering algorithms.

**SC **^37^: This is a single-view spectral clustering algorithm which can deal with nonlinear structural data well. The results of the best view have been extracted in this article.

**Co-Reg **^38^: Co-Reg searches for the consistency graph of multiple views by co-regularizing clustering assumptions so that clustering performance is better than that of a single view.

**AMGL**^39^: AMGL automatically assigns a weight to each view without additional parameters, which can be well used for multi-view clustering and semi-supervised classification tasks.

**SwMC **^25^: The algorithm imposes Laplacian rank constraint on the unified similarity matrix and automatically learns the weights.

**GFSC **^40^: The algorithm utilizes a self-representation method to construct the representation matrix of each view, which is then fused and clustering results are obtained using a single-view spectral clustering algorithm.

**MVGL **^41^: The algorithm learns the affinity graph of each view and fuses it into a high-quaity unified graph.

**GMC **^26^: GMC integrates the affinity graph of each view into a unified graph and imposes Laplacian rank constraint on the unified graph to obtain the clustering result directly.

**AWP**^42^: AWP extends the spectral rotation method in spectral clustering and combines it with Procrustes Analysis to automatically assign the weight of each view.

**S-MVSC **^43^: S-MVSC learns consistent sparse unified graph through multiple views, which has fast clustering speed and can achieve prosperous clustering results.

**MCDCF **^44^: MCDCF applies a matrix decomposition method (deep matrix decomposition) to multi-view clustering and integrates them into a unified framework.

The results of part of the comparison algorithms refer to Reference^45^. Each algorithm was run 30 times for each dataset, recording its mean and standard deviation, with the best results in italics and the second-best in bold. Tables 2, 3, 4, 5, 6, 7 lists the clustering results of IMVSCP and other comparison algorithms. Before the experiment, the number of neighbors of IMVSCP algorithm needs to be adjusted. In this paper, the number of neighbors for BBCSport, ORL, MSRC, Mfeat, NUS and 3-source datasets was set to 80, 8, 33, 60, 35, 100, respectively.

**Table 2:** Experimental results (mean standard deviation) on the BBCSport dataset.

Method	Acc	NMI	F-score	ARI	Precision	Recall
SC	0.431±0.000	0.178±0.000	0.398±0.003	0.091±0.022	0.282±0.013	0.688±0.087
Co-Reg	0.567±0.052	0.421±0.029	0.481±0.040	0.290±0.050	0.426±0.029	0.552±0.060
AMGL	0.385±0.010	0.045±0.005	0.385±0.001	0.029±0.011	0.251±0.005	0.831±0.045
SwMC	0.382±0.000	0.084±0.001	0.391±0.001	0.019±0.001	0.246±0.000	*0.960*±*0.002*
GFSC	0.579±0.000	0.238±0.000	0.453±0.000	0.178±0.000	0.323±0.000	0.756±0.000
MVGL	0.434±0.000	0.182±0.000	0.402±0.000	0.050±0.000	0.259±0.000	0.895±0.000
GMC	0.809±0.000	0.701±0.000	0.759±0.000	0.667±0.000	0.656±0.000	0.902±0.000
AWP	0.634±0.028	0.517±0.019	0.552±0.024	0.408±0.027	0.542±0.010	0.564±0.043
S-MVSC	**0.852**±**0.086**	**0.821**±**0.051**	**0.845**±**0.055**	**0.798**±**0.072**	**0.853**±**0.059**	0.839±0.058
MCDCF	0.774±0.073	0.683±0.054	0.738±0.056	0.634±0.087	0.638±0.104	0.892±0.043
IMVSCP	*0.972*±*0.000*	*0.910*±*0.000*	*0.942*±*0.000*	*0.924*±*0.000*	*0.948*±*0.000*	**0.936**±**0.000**

Significant values are in [bold/italics].

**Table 3:** Experimental results (mean standard deviation) on the ORL dataset.

Method	Acc	NMI	F-score	ARI	Precision	Recall
SC	0.797±0.033	0.929±0.010	0.768±0.031	0.762±0.032	0.711±0.039	0.836±0.026
Co-Reg	**0.808**±**0.008**	0.933±0.003	**0.778**±**0.008**	**0.772**±**0.008**	**0.723**±**0.010**	0.842±0.005
AMGL	0.635±0.055	0.888±0.019	0.547±0.090	0.533±0.094	0.420±0.098	0.811±0.017
SwMC	0.739±0.051	0.903±0.028	0.511±0.115	0.495±0.120	0.373±0.113	0.853±0.033
GFSC	0.575±0.000	0.753±0.000	0.346±0.000	0.324±0.000	0.231±0.000	0.690±0.000
MVGL	0.788±0.000	*0.937*±*0.000*	0.715±0.000	0.707±0.000	0.602±0.000	*0.880*±*0.000*
GMC	0.765±0.000	0.891±0.000	0.596±0.000	0.584±0.000	0.454±0.000	**0.869**±**0.000**
AWP	0.748±0.035	0.884±0.011	0.675±0.033	0.667±0.033	0.647±0.034	0.705±0.033
S-MVSC	0.753±0.034	0.897±0.009	0.701±0.028	0.694±0.028	0.646±0.037	0.767±0.019
MCDCF	0.714±0.041	0.858±0.017	0.627±0.048	0.617±0.050	0.554±0.066	0.728±0.020
IMVSCP	*0.870*±*0.000*	**0.935**±**0.000**	*0.832*±*0.000*	*0.828*±*0.000*	*0.810*±*0.000*	0.855±0.000

Significant values are in [bold/italics].

**Table 4:** Experimental results (mean standard deviation) on the MSRC dataset.

Method	Acc	NMI	F-score	ARI	Precision	Recall
SC	0.677±0.069	0.600±0.048	0.566±0.057	0.495±0.067	0.557±0.061	0.576±0.053
Co-Reg	0.663±0.128	0.563±0.130	0.551±0.128	0.476±0.149	0.539±0.125	0.563±0.131
AMGL	0.714±0.061	0.674±0.030	0.603±0.037	0.529±0.048	0.540±0.052	0.687±0.026
SwMC	0.782±0.063	0.725±0.061	0.679±0.070	0.623±0.084	0.635±0.083	0.732±0.059
GFSC	0.676±0.000	0.703±0.000	0.637±0.000	0.569±0.000	0.560±0.000	0.740±0.000
MVGL	**0.833**±**0.000**	0.713±0.000	0.693±0.000	0.642±0.000	0.676±0.000	0.711±0.000
GMC	0.748±0.000	**0.742**±**0.000**	0.697±0.000	0.640±0.000	0.612±0.000	*0.809*±*0.000*
AWP	0.775±0.081	0.667±0.053	0.660±0.073	0.604±0.087	0.650±0.083	0.672±0.064
S-MVSC	0.831±0.036	*0.744*±*0.013*	**0.728**±**0.022**	**0.683**±**0.026**	**0.713**±**0.034**	0.744±0.009
MCDCF	0.754±0.035	0.673±0.023	0.638±0.023	0.577±0.026	0.617±0.020	0.660±0.027
IMVSCP	*0.848*±*0.000*	0.731±0.000	*0.738*±*0.000*	*0.695*±*0.000*	*0.729*±*0.000*	**0.747**±**0.000**

Significant values are in [bold/italics].

**Table 5:** Experimental results (mean standard deviation) on the Mfeat dataset.

Method	Acc	NMI	F-score	ARI	Precision	Recall
SC	0.717±0.000	0.661±0.000	0.616±0.038	0.573±0.042	0.609±0.039	0.627±0.037
Co-Reg	0.743±0.082	0.733±0.035	0.685±0.059	0.649±0.066	0.662±0.070	0.710±0.047
AMGL	0.755±0.000	0.826±0.000	0.742±0.000	0.710±0.000	0.677±0.000	0.820±0.000
SwMC	0.845±0.025	*0.896*±*0.020*	0.838±0.027	0.818±0.030	0.776±0.033	*0.910*±*0.018*
GFSC	0.814±0.000	0.757±0.000	0.719±0.000	0.687±0.000	0.698±0.000	0.742±0.000
MVGL	**0.928**±**0.000**	**0.882**±**0.000**	**0.862**±**0.000**	**0.846**±**0.000**	**0.852**±**0.000**	0.872±0.000
GMC	0.836±0.000	0.851±0.000	0.811±0.000	0.788±0.000	0.747±0.000	**0.887**±**0.000**
AWP	0.763±0.082	0.721±0.053	0.655±0.087	0.616±0.097	0.652±0.088	0.657±0.086
S-MVSC	0.792±0.066	0.852±0.032	0.779±0.065	0.751±0.075	0.708±0.085	0.871±0.024
MCDCF	$\sim$	$\sim$	$\sim$	$\sim$	$\sim$	$\sim$
IMVSCP	*0.931*±*0.000*	0.867±0.000	*0.869*±*0.000*	*0.854*±*0.000*	*0.867*±*0.000*	0.871±0.000

Significant values are in [bold/italics].

‘$\sim$’ indicates that the running time of the algorithm exceeds one hour, and the following is the same

**Table 6:** Experimental results (mean standard deviation) on the NUS dataset.

Method	Acc	NMI	F-score	ARI	Precision	Recall
SC	0.221±0.008	0.093±0.003	0.131±0.003	0.050±0.004	0.127±0.004	0.136±0.003
Co-Reg	0.264±0.031	0.133±0.025	0.157±0.012	0.078±0.014	0.153±0.013	0.161±0.011
AMGL	0.234±0.007	**0.161**±**0.002**	0.162±0.002	0.055±0.003	0.119±0.003	0.254±0.024
SwMC	0.147±0.011	0.107±0.014	0.154±0.002	0.005±0.002	0.086±0.001	*0.802*±*0.088*
GFSC	0.253±0.000	0.134±0.000	0.154±0.000	0.071±0.000	0.142±0.000	0.167±0.000
MVGL	0.154±0.000	0.117±0.000	0.156±0.000	0.009±0.000	0.087±0.000	**0.756**±**0.000**
GMC	0.186±0.000	0.094±0.000	0.158±0.000	0.018±0.000	0.092±0.000	0.555±0.000
AWP	0.276±0.027	0.097±0.011	0.140±0.011	0.061±0.012	0.138±0.011	0.141±0.010
S-MVSC	**0.294**±**0.006**	**0.161**±**0.005**	**0.172**±**0.003**	**0.096**±**0.003**	**0.169**±**0.002**	0.176±0.005
MCDCF	$\sim$	$\sim$	$\sim$	$\sim$	$\sim$	$\sim$
IMVSCP	*0.298*±*0.000*	*0.167*±*0.000*	*0.177*±*0.000*	*0.101*±*0.000*	*0.173*±*0.000*	0.181±0.000

Significant values are in [bold/italics].

**Table 7:** Experimental results (mean standard deviation) on the 3-sources dataset.

Method	Acc	NMI	F-score	ARI	Precision	Recall
SC	0.555±0.000	0.469±0.000	0.487±0.024	0.349±0.031	0.536±0.035	0.448±0.027
Co-Reg	0.551±0.031	0.493±0.017	0.468±0.013	0.321±0.018	0.504±0.031	0.439±0.027
AMGL	0.392±0.064	0.190±0.065	0.345±0.019	0.014±0.045	0.240±0.023	0.628±0.086
SwMC	0.396±0.049	0.189±0.051	0.350±0.009	0.002±0.021	0.232±0.009	0.719±0.049
GFSC	0.450±0.000	0.327±0.000	0.343±0.000	0.075±0.000	0.275±0.000	0.455±0.000
MVGL	0.408±0.000	0.202±0.000	0.343±0.000	0.007±0.000	0.229±0.000	0.677±0.000
GMC	0.757±0.000	0.597±0.000	0.659±0.000	0.528±0.000	0.552±0.000	*0.817*±*0.000*
AWP	0.294±0.007	0.083±0.006	0.257±0.006	0.010±0.005	0.226±0.003	0.298±0.016
S-MVSC	0.707±0.054	0.676±0.031	0.651±0.050	0.562±0.062	**0.739**±**0.055**	0.583±0.049
MCDCF	**0.776**±**0.026**	**0.684**±**0.026**	**0.721**±**0.030**	**0.627**±**0.047**	0.675±0.058	0.776±0.016
IMVSCP	*0.793*±*0.000*	*0.701*±*0.000*	*0.776*±*0.000*	*0.708*±*0.000*	*0.768*±*0.000*	**0.785**±**0.000**

Significant values are in [bold/italics].

- The IMVSCP algorithm is an improvement based on the spectral clustering algorithm. It can be seen from the experimental results that in terms of algorithm accuracy, the IMVSCP algorithm on BBCSport, ORL, MSRC, Mfeat, NUS and 3-sources datasets are 0.541, 0.073, 0.171, 0.214, 0.077 and 0.238 higher than the spectral clustering algorithm (SC), respectively. This proves that multi-view clustering performs better than single-view clustering because it can well integrate information from multiple views.
- Co-Reg, AMGL, SWMC, MVGL, AWP and S-MVSC all construct the initial similarity matrix of each view in a static way, while IMVSCP constructs the optimal similarity matrix of each view in a dynamic way. Therefore, the clustering performance of IMVSCP is generally better than these algorithms. For example, the IMVSCP algorithm is 0.405, 0.587, 0.59, 0.538, 0.338 and 0.12 higher than the above algorithms in the clustering accuracy on the BBCSport dataset.
- GFSC algorithm uses self-representation method to construct affinity matrix of each view, and finally utilizes post-processing operation (k-means) to obtain clustering result. The experimental results indicate that IMVSCP algorithm is superior to GFSC algorithm in each index, which demonstrates the effectiveness of the method of dynamically obtaining the similarity matrix of each view and the method of spectral clustering combined with symNMF adopted in this paper.
- The standard deviation of IMVSCP algorithm is 0, indicating that the clustering result does not change without considering the times of calculation when the number of neighbors is fixed. This verifies the computational stability of the IMVSCP algorithm.
- In general, the clustering performance of multi-view clustering algorithm is greatly affected by the quality of similarity matrix of each view and the method of obtaining clustering results. Compared with these state-of-the-art algorithms, IMVSCP algorithm has the advantage of dynamically obtaining high-quality similarity matrix of each view, and combining spectral clustering algorithm and symmetric nonnegative matrix factorization method to output clustering results directly, so as to avoid the information loss caused by the second operation.

**Figure 3:**
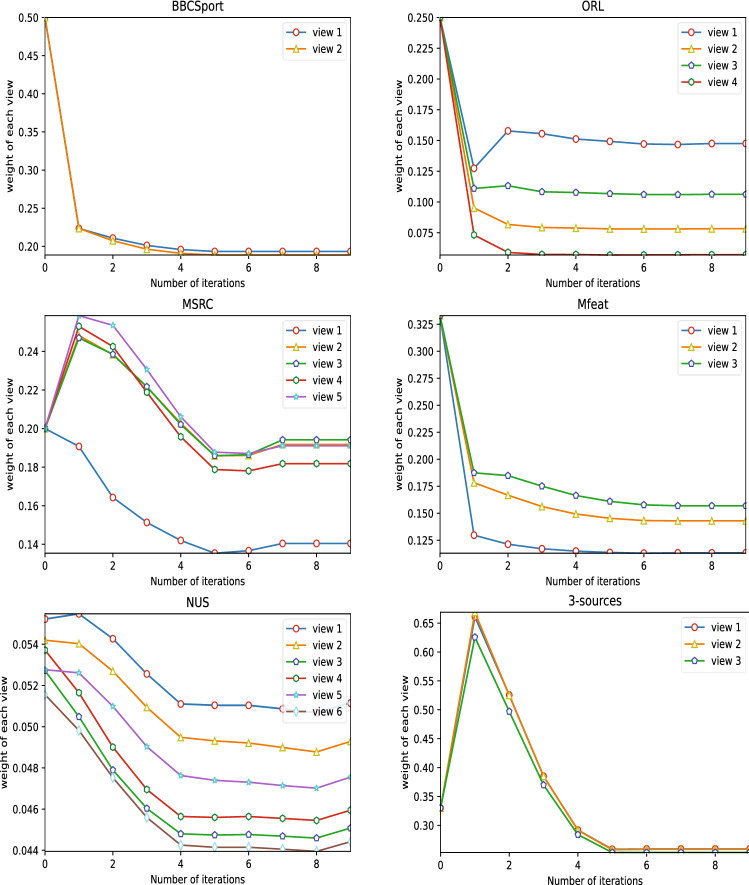
The weight change of each view in the optimization process by algorithm 1.

In the optimization of the similarity matrix of each view by algorithm 1, it is necessary to automatically assign weights to each view according to the quality of each view, so as to obtain the optimal similarity matrix of each view. Figure 3 shows the weight change of each view in the optimization process by algorithm 1. It can be seen from Fig. 3 that the weight of each view of BBCSport and 3 source datasets are roughly the same, indicating that the quality of each view is not much different, while the quality of the remaining four datasets is uneven.

### Ablation study

For numerous graph-based multi-view clustering, since the Laplacian rank constraint is applied to the unified matrix to make the unified matrix have block structure and can directly output the clustering results, the noise and redundant information cannot be well removed. On the other hand, in order to verify the impact of the optimized similarity matrix of each view in this paper on the improvement of clustering performance, ablation experiments were carried out.

**Figure 4:**
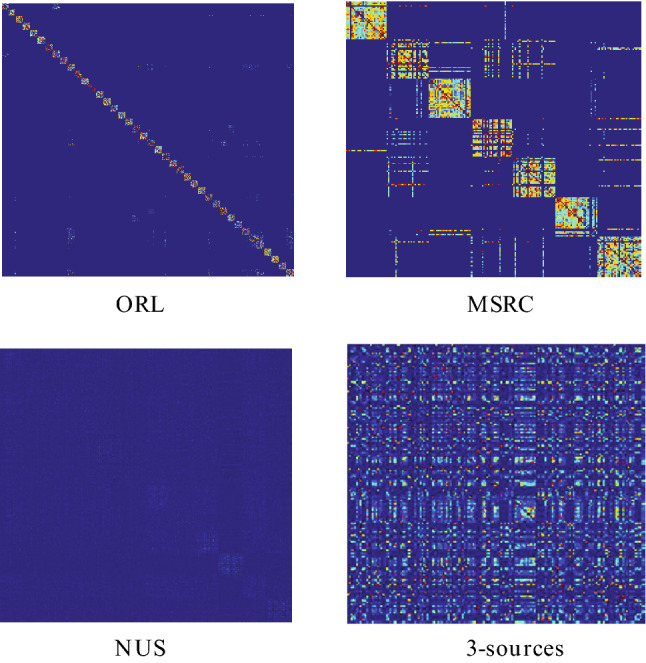
Visualize the unified matrix obtained by IMVSCP-1.

Firstly, we utilized IMVSCP-1 to output the clustering results directly by means of the unified matrix *P* obtained by algorithm 1 because *P* was imposed Laplacian rank constraint. In addition, We define IMVSCP-2 to obtain the similarity matrix for each view using the k nearest neighbors of the graph rather than the method proposed in this paper to dynamically obtain the similarity matrix of each view. We designed the IMVSCP-3 algorithm to remove the rank constraint on the unified matrix *P* to verify the effect of rank constraint on the clustering performance. Four algorithms were run under the identical experimental conditions. Table 8 shows the comparison results of IMVSCP, IMVSCP-1, IMVSCP-2 and IMVSCP-3 on ORL, MSRC, NUS and 3-sources datasets, with the best results in bold. Figure 4 visualizes the unified matrix obtained by IMVSCP-1.

**Table 8:** The comparison results of IMVSCP, IMVSCP-1, IMVSCP-2 and IMVSCP-3 on ORL, MSRC, NUS and 3-sources datasets.

		IMVSCP-1	IMVSCP-2	IMVSCP-3	IMVSCP
ORL	Acc	0.765	0.825	0.765	**0.870**
	Precision	0.454	0.728	0.454	**0.810**
	ARI	0.584	0.765	0.584	**0.828**
MSRC	Acc	0.804	0.791	0.757	**0.848**
	Precision	0.672	0.690	0.626	**0.729**
	ARI	0.685	0.662	0.632	**0.695**
NUS	Acc	0.186	0.297	0.157	**0.298**
	Precision	0.091	0.160	0.087	**0.173**
	ARI	0.011	0.094	0.009	**0.101**
3-sources	Acc	0.757	0.781	0.787	**0.793**
	Precision	0.552	0.704	0.621	**0.768**
	ARI	0.528	0.643	0.603	**0.708**

Significant values are in [bold].

The clustering performance of IMVSCP algorithm is better than that of IMVSCP-1 and IMVSCP-2 algorithm from Table 8. It can be seen from Fig. 4 that for ORL and MSRC datasets, block structures can be seen, but there is still a lot of noise around. Nevertheless, for NUS and 3-sources datasets, the block structures are not visible. This indicates that IMVSCP-1 algorithm cannot deal with noise data well and its application range is small. In addition, the clustering performance of IMVSCP is better than that of IMVSCP-2, which fully demonstrates that using a dynamically optimized similarity matrix for each view can obtain better clustering results than using the static method. Ablation study verify that IMVSCP-1 and IMVSCP-2 algorithms are complementary and indispensable. Furthermore, although the clustering performance of IMVSCP-3 algorithm is the same as that of IMVSCP-1 on the dataset ORL and higher than that of IMVSCP-1 on the dataset 3Sources, in general, the clustering performance of IMVSCP-3 without rank constraint is worse than that of IMVSCP-1.

### Visual analysis

**Figure 5:**
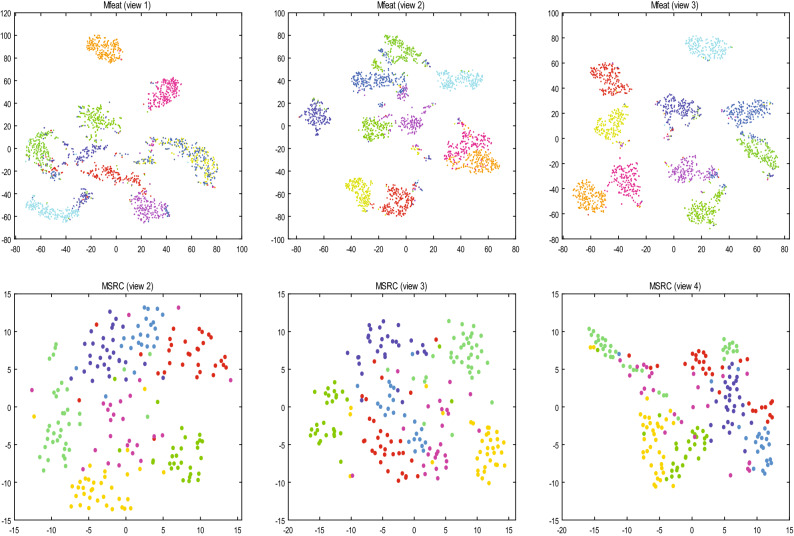
The t-SNE experiments on Mfeat and MSRC datasets.

In order to make the clustering results of IMVSCP algorithm more intuitive to be verified, t-SNE experiments on 3 views of Mfeat and MSRC datasets were performed. The t-SNE experiments on these two datasets are shown in Fig. 5. From the visualization results of Mfeat dataset, the dark blue and light green points in view 1 are not well separated but are well parted in other views. Combined with the experiment of view weight assignment of Mfeat dataset in Fig. 3, the quality of view 1 of Mfeat is poor, so it is assigned a lower weight, which proves the effectiveness of IMVSCP to optimize each similarity matrix. The clustering results of view 2, 3, 4 of MSRC dataset are similar, so it can be seen from Fig. 3 that the weights of these three views are not significantly different.

### Convergence and time consumption analysis

**Figure 6:**
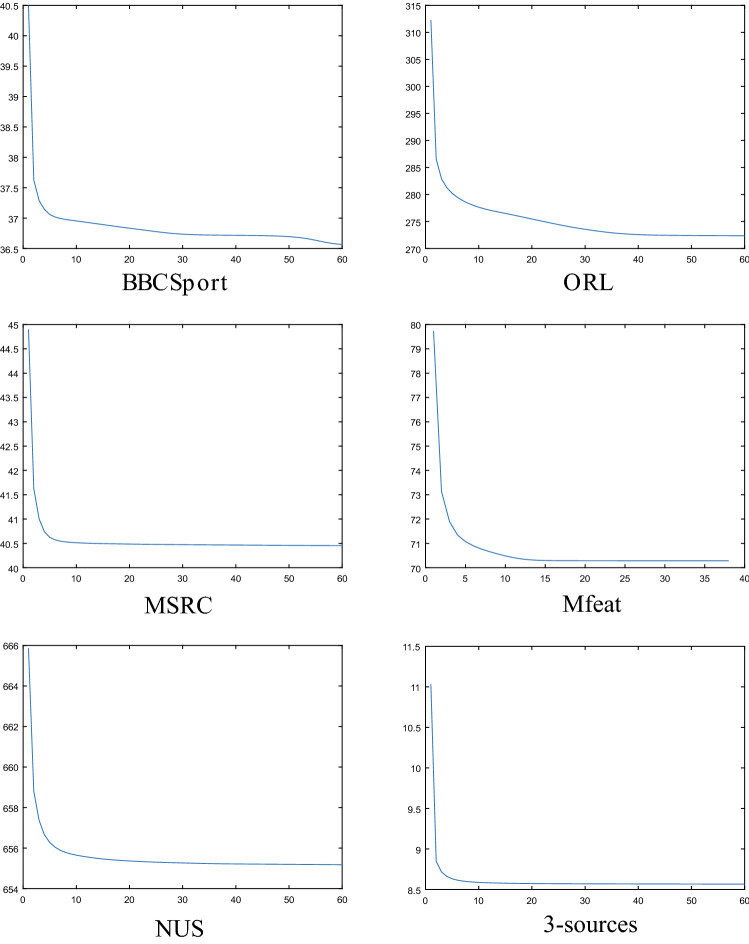
The change in the value of the objective function of algorithm 2.

To further verify the convergence of IMVSCP algorithm, the variation of the objective function value of algorithm 2 with the number of iterations is shown in Fig. 6. It is proved that the convergence rate of algorithm 2 is fast enough to converge within 60 iterations. Table 9 shows the comparison of running time of some state-of-the-art algorithms on six datasets. As can be seen from Table 9, although the IMVSCP algorithm first generates the similarity matrix of each view dynamically and iteratively, and then the spectral clustering algorithm is combined with the symmetric nonnegative matrix factorization algorithm to generate clustering results, the running time of IMVSCP is not longer than other state-of-the-art algorithms on the whole.

**Table 9:** Comparison of running time of some algorithms (seconds).

	BBCSport	ORL	MSRC	Mfeat	NUS	3-sources
SwMC	0.3607	0.6774	0.309	137.5248	180.7202	0.4245
GFSC	2.4458	2.1415	0.8174	71.4697	184.8681	0.9066
GMC	10.6281	0.6502	0.4242	19.3592	41.2571	0.3337
MCDCF	39.3723	31.8589	6.5644	$\sim$	$\sim$	4.897
IMVSCP	79.0186	2.1023	0.8551	33.0712	80.2132	0.752

### Impact of different number of neighbors

**Figure 7:**
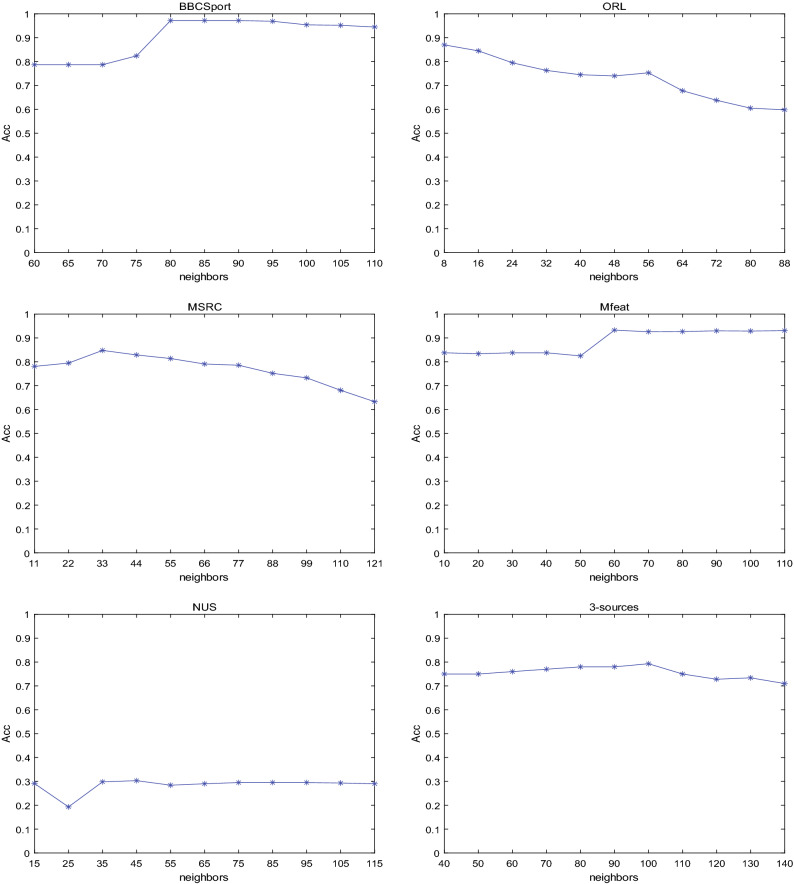
Impact of the change in the number of neighbors on Acc.

We selected an appropriate range and step size for each dataset to verify the impact of the number of neighbors on the clustering performance. Figure 7 shows the impact of the change in the number of neighbors on Acc on the six datasets. It can be seen from Fig. 7 that the change of the number of neighbors in a certain range has a certain impact on the clustering performance. In future research, we will try to avoid the influence of the number of neighbors on the clustering performance.

## Discussion

In previous studies, reference^26^ adopted the method of imposing Laplacian rank constraint on the unified matrix to directly output the clustering results, without this part of algorithm 2 in this paper. Reference^43^ uses the k-NN method to construct the similarity matrix of each view. IMVSCP combines the advantages of the two algorithms to improve the clustering performance, which is verified by ablation study. In the ablation study, algorithm 1 was utilized only to generate clustering results by removing algorithm 2, and then k-NN algorithm was used to construct the initial similarity matrix of each view and algorithm 2 was used to generate clustering results. The ablation study results verified that algorithm 1 and algorithm 2 complement each other and are indispensable.

However, the number of neighbors needs to be set in advance in the IMVSCP algorithm, which will affect the robustness of the algorithm. For example, toward the six datasets BBCSport, ORL, MSRC, Mfeat, NUS and 3sources used in this article, the optimal number of neighbors is 80,8,33,60,35,100, respectively. Therefore, we will focus on this problem in the future. The method proposed in this paper has a wide range of application scenarios. For example, in the cluster analysis of aerial image data, each scene and object can be accurately identified. It can also play a role in the field of medical impact analysis. In addition, the proposed method can also be used to solve problems related to the Internet of Things^46^.

## Conclusion

In this paper, an improved multi-view spectral clustering based on tissue-like P systems (IMVSCP) was proposed to construct a high-quality similarity matrix for each view and improve clustering performance. Firstly, the similarity matrix of each view is optimized in a dynamic way to obtain high-quality similarity matrix of each view. Then, spectral clustering and nonnegative symmetric matrix factorization are combined to directly output the clustering results without secondary operation. On the other hand, IMVSCP is combined with tissue-like P system to make it run in the framework of tissue-like P system, which improves the efficiency of the algorithm. Extensive experiments verify that IMVSCP algorithm is superior to the state-of-the-art multi-view clustering algorithms and single-view spectral clustering algorithm in clustering performance.

## Data Availability

This article uses six datasets, which can be obtained as follows: BBCSport: http://mlg.ucd.ie/datasets/ ORL: https://cam-orl.co.uk/facedatabase.html MSRC: https://www.researchgate.net/publication/335857675 Mfeat: http://archive.ics.uci.edu/ml/datasets/Multiple+Features NUS: https://lms.comp.nus.edu.sg/wp-content/uploads/2019/research/nuswide/NUS-WIDE.html 3sources: http://mlg.ucd.ie/datasets/3sources.html.
